# Supplementary material for: Are carbon emissions trading and green financial instruments synergistic? -Comprehensive quantitative research based on content analysis
Source: PLoS One. 2024 Mar 7;19(3):e0298601. doi: 10.1371/journal.pone.0298601 (PMC10919601; doi:10.1371/journal.pone.0298601)
Supplement: S1 Data — (ZIP) [file pone.0298601.s001.zip › Data/地级市Z_F工作报告.zip!/║╙▒▒/╨╧╠¿╩╨/└┤╫╘╣1⁄2╓┌║┼í╛┬φ┐╦╩2╛▌═°í┐_2018.docx]

各位代表：

　　现在，我代表邢台市人民政府，向大会作政府工作报告，请予审议，并请各位政协委员和列席会议的同志提出意见。

　　一、2017年工作回顾

　　2017年，全市人民在党的十九大精神指引下，坚持以习近平新时代中国特色社会主义思想为指导，增强“四个意识”，坚定“四个自信”，坚持稳中求进工作总基调，贯彻落实新发展理念，深入推进供给侧结构性改革，在省委、省政府和市委的正确领导下，在市人大、市政协的监督支持下，改革创新，克难攻坚，一些领域实现突破，各项事业长足发展，较好完成了市十五届人大一次会议确定的目标任务，为决胜全面建成小康社会，加快建设新时代经济强市、美丽邢台奠定了坚实基础。

　　——综合实力稳步提升。以新理念引领新发展，以增比进位激励发展干劲，以工作提标推动发展提速，生产总值完成2236.4亿元，增长7%，突破2000亿元大关；全部财政收入232.4亿元，增长19.5%，突破200亿元大关；一般公共预算收入126.7亿元，增长13.7%；规模以上工业增加值773.3亿元，增长6.4%；固定资产投资2133.3亿元，增长5.3%；实际利用外资6亿美元，增长10.9%；出口总值110.5亿元，增长24.3%；社会消费品零售总额1076.2亿元，增长11%。全市金融机构存贷款余额分别达到3746亿元、2325亿元，分别增长12.3%、16.5%。主要指标增速均高于全省平均水平，一般公共预算收入、规模以上工业增加值增速全省第一。

　　——产业结构持续优化。以供给侧结构性改革为主线，扎实做好“三去一降一补”各项工作，坚决去、主动调、加快转，推进新旧动能转换。压减炼钢产能72万吨、煤炭482万吨、焦炭60万吨、玻璃260万重量箱、小火电7台9万千瓦，提前超额完成省“6643”下达任务。装备制造业增加值占规模以上工业比重30.1%，提高2.4个百分点；规模以上高新技术产业增加值增长16.6%，高于规模以上工业增速10.2个百分点。粮食总产475.1万吨，农业产业化经营率达到70%。旅游业总收入增长30.9%，服务业对经济增长贡献率达到58.3%。三次产业结构比由13.7︰46.8︰39.5调整为12.2︰47.9︰39.9。

　　——基础设施日臻完善。坚持以人民为中心的发展思想，做优老城、做大新城、做强县城、做美村镇，中心城区总投资1500亿元的9大类225项城建工程顺利推进。投资40多亿元、全长79.8公里的大外环全面开工、部分通车，城市框架拉大4倍；市人民医院新院区、邢台中学、市规划馆、莲池大街立交桥等重点工程加快实施。成功创建第一届河北省文明城市，取得河北省第三届园博会举办权。邢和铁路、太行山高速等重大基础设施工程加快建设。

　　——民生保障不断加强。始终将增进民生福祉作为发展的根本目的，财政用于民生支出340.3亿元，占一般公共预算支出的81.1%。城镇、农村居民人均可支配收入分别达到26179元、10999元，分别增长9.5%、9.9%。新开工棚户区改造住房2.25万套，基本建成2.23万套。理顺了市区中小学、幼儿园管理体制。企业职工养老保险中断缴费率下降5.03个百分点。城镇登记失业率3.45%，新增城镇就业7.78万人。

　　一年来，我们主要抓了以下五方面工作：

　　（一）加快产业转型升级，着力提高质量效益。全市牢固树立“两个不能”工作导向，成立四个市级专业招商部和重点企业服务办公室，全力推进高质量的投资和项目建设。省重点项目完成投资108.7亿元，超年计划14个百分点；全市亿元以上施工项目完成投资1293.3亿元，增长16.4%；晶澳太阳能电池等81个项目投产，其中24个项目当年开工、当年投产；市重点在建项目中，高新技术和服务业项目占比45.3%，提高9.5个百分点。投资80亿元的中钢邢机搬迁改造项目扎实推进，将打造全球领先的轧辊研发生产、高端特种锻造锻件生产、全国焦炉维保“三大中心”，原址将建设双创中心、精品街区和城市记忆公园。东风汽车团队进驻长征汽车，共同研发的渣土车开始试生产；御捷车业与长城汽车合作的年产15万台新能源乘用车项目开工建设。用工业设计提升特色产业，清河羊绒、隆尧三厦等企业走在全市前列。平乡童车、临西轴承入选全国第四批产业集群区域品牌建设试点。农业供给侧结构性改革取得新进展，新增省级现代农业园区8家，金沙河小麦精加工、今麦郎饮品、玉锋淀粉等食品工业扩能升级项目部分投产，全国春季农业生产暨现代农业产业园建设工作会议在我市观摩，桥西区被列为全国农村集体产权制度改革试点，隆尧县获评全国首个食品工业品牌创新发展基地，南和县成功创建首批国家农村产业融合发展示范园。

　　（二）提升城市品质价值，着力建设美好家园。强力推进中心城区建设三年攻坚，邢石大道、滨江路、东华路大修全线通车，建设大街、新华北路等16条断头路贯通。邢东新区总体规划、土地利用规划高标准完成，路网管网、生态公园等46项重点工程完成投资56.9亿元。实施市区供热三年攻坚，86条道路有序开挖，三年工程两年完成，市区集中供热率达到97.66%。坚持“一步到位、适度超前”，实施污水处理能力提升三年攻坚，完成20条街道雨污分流。深入开展“一区三边”集中整治，以拆促建、以拆促提，拆除743.5万平方米，市本级拆迁量全省第一，拆迁地块优先用于绿化和公共设施建设，市区新增乔木量是前两年总和的5.8倍，新增街头游园30处。投资1.67亿元改造老旧小区442个，逐一解决了200项房地产遗留问题。新改建市区公厕146座，新投放共享单车3000辆。深入实施县城建设三年攻坚，完成城建重点工程投资160.8亿元，南和县被命名为国家卫生县城，威县、任县入选国家园林县城，新增柏乡牡丹小镇、清河汽摩小镇2个全国特色小镇。

　　（三）持续深化改革创新，着力增强活力动力。扎实推进“放管服”改革，向县市区“点菜”式下放383项行政权力。市行政审批局挂牌运行，29个部门、331项事项统一划转、集中办理，实现51枚印章合一。出台全省首张证照清单，全面推行“二十五证合一”，453项审批服务事项实现“最多跑一次”。新增市场主体8.95万户，同比增长16.13%。整合建设检验检测、应急指挥、便民服务“三个中心”，方便企业群众，节约公共资源。集约用地做法全国推广，“多规合一”、环保机构垂直管理、城市综合执法、国有企业“三供一业”等改革走在全省前列，农业农村、供销社等各项改革有序推进。成功入选我省唯一一家全国PPP创新工作重点城市，设立了蓝天基金等5支基金。深入实施创新驱动战略，争取省以上科技项目115个，新增省级院士工作站3家、产业技术研究院1家，新认定高新技术企业60家、科技型中小企业1573家，威县、沙河被列为省第二批创新型县市区试点，新增国家级星创天地2家、省级农业科技园区4家。

　　（四）科学精准铁腕治霾，着力改善生态环境。面对年初PM2.5平均浓度一度上升44.9%的严峻形势和日益明晰的环保红线，全市上下牢固树立“绿水青山就是金山银山”理念，以空前的决心和力度开展大气污染综合治理。关停拆除“散乱污”企业5459家，彻底解决了困扰市区周边30多年的小板厂“脏乱差”问题，以整治落后的“减法”换来了动能转换的“加法”和经济效益、社会效益、生态效益的“乘法”；拆除邢钢、德龙4座在产竖炉，结束了我市钢铁企业球团工序生产的历史；2384台10蒸吨及以下燃煤锅炉全省率先清零、集中存放，防止了二次流入市场；完成气（电）代煤16万户，千方百计保证气源，采暖季优先用于民生；投资3.7亿元购置更换清洁能源公交车250辆、作业车675台，实现市区公共服务清洁作业；严格“以克论净”，531个建筑工地全部落实“六个百分之百”，主城区清洁湿扫全覆盖，机械化清扫率达到86.6%。采取最严秋冬防措施，邢钢4座高炉停产3座，德龙3座高炉停产2座，旭阳9条生产线停产5条，建滔4条生产线停产2条，玻璃生产线关停19条。通过一天一天努力，一微克一微克争取，全年PM2.5平均浓度下降8.25%，空气质量综合指数下降3.2%，超额完成省定任务，保持了2014年以来的“四连降”，实现了区域空气质量最好。全面落实河长制，加快市域河流退污还清，市污水处理二厂在保证质量的同时昼夜施工、全力推进。顺利通过全国水生态文明城市验收。争取到国家储备林试点城市，创新建设模式，利用长期低息资金进行造林绿化，全市新增造林面积54.62万亩，森林覆盖率提高1.23个百分点。

　　（五）聚焦补齐民生短板，着力增进人民福祉。实施精准扶贫、精准脱贫，全面完成建档立卡“回头看”，农村低保标准提高到3300元/年，威县“金鸡扶贫”模式被列为中央政治局集体学习时的参阅材料，7.75万贫困人口稳定脱贫，贫困发生率由2.85%下降到1.65%。灾后重建315个省以上补助资金项目全部完工，首创重建项目指标评估体系并在全省推广。最大力度保障冬季供暖，开展拉网式排查，及时发现解决问题，确保每户群众安全温暖过冬。实施市区基础教育三年攻坚，新改扩建学校7所，威县、邢台县等7个县市通过国家义务教育发展基本均衡县验收。职业院校省级骨干特色专业达到29个，在第44届世界技能大赛中，我市选手代表河北省参赛实现奖牌零的突破。社保卡金融诊疗“一卡通”在全省率先启动，巨鹿“医养结合”模式得到国务院领导肯定批示。第二届国际公路自行车赛和首届旅发大会成功举办，邢台知名度、美誉度不断提升。话剧《李保国》、河北梆子《吕玉兰》、电影《有家》等作品入选省“五个一工程”奖。深入开展“创净土”等严打整治集中行动，平安邢台建设全面加强。食品药品安全工作全省领先，安全生产形势持续稳定，双拥共建、民族宗教、外事侨务、邮政通信、新闻广电、审计统计、质量安全、人防地震、方志档案、老龄妇幼、残疾人事业、援疆援藏等工作都取得新成绩。

　　一年来，我们坚决落实全面从严治党的战略部署，不断加强政府自身建设。法治政府建设深入推进，严格落实政务“五公开”，市级机关内设机构改革全部完成。自觉接受人大法律监督、工作监督和政协民主监督，办理人大议案建议434件，政协提案789件。加大廉政建设和反腐败工作力度，坚持不懈纠正“四风”，查处违反中央八项规定精神典型问题701起，党政纪处分505人。政府职能转变、作风建设取得扎实成效。

　　各位代表，一年来的艰辛努力不同寻常，每一项成绩的取得都极为不易。这是习近平新时代中国特色社会主义思想引航定向、伟大实践的结果，是省委、省政府和市委正确领导的结果，是人大、政协和社会各界监督支持的结果，是全市人民团结拼搏的结果。在此，我代表市政府，向全市人民，向人大代表、政协委员、各民主党派、工商联、无党派人士和人民团体，向驻市部队、武警官兵、政法干警和驻市各单位、各企业，向所有关心支持邢台发展的各位老领导、老同志以及海内外朋友，表示崇高的敬意和衷心的感谢！

　　看到成绩的同时，我们也清醒认识到，目前邢台仍处于转型升级、爬坡过坎的关键时期，许多深层次的矛盾和问题尚未解决，我们的工作还有很多不足，发展仍然面临诸多困难和挑战。一是经济实力弱，总量小、人均低、欠发达的市情还没有根本改变。二是产业层级总体偏低，动能转换不快的矛盾依然突出。三是城乡建设总体滞后，城市经济还不强，乡村振兴任务艰巨，教育、医疗、住房等方面与群众期盼还有不少差距。四是环境治理形势依然严峻，大气污染防治任务重、攻坚难度大，水、土壤污染治理刻不容缓。五是营商环境还不优，一些政府部门和工作人员不担当不作为、效率低落实慢，一些领域“四风”问题和消极腐败现象仍有发生。2017年《政府工作报告》确定的177项重点工作、重大工程、民生实事，还有14项没有完成。面对高质量发展要求，如果高消耗、高污染、高排放产业压不下去，环境资源将难以为继；如果新兴产业顶不上来，经济增长将难以为继；如果风险隐患处理不好，稳定发展将难以为继；如果质量效益提不起来，民生保障将难以为继。

　　各位代表，实现高质量发展是更大的挑战，也是我们义不容辞的历史责任。我们将切实增强忧患意识，牢固树立问题导向，知危图安，担当实干，一个问题一个问题地解决，一项任务一项任务地落实，不忘初心、继续前进，撸起袖子加油干，奋力谱写伟大复兴中国梦的邢台篇章！

　　二、2018年工作安排

　　2018年是全面贯彻党的十九大精神的开局之年，是改革开放40周年，是决胜全面建成小康社会、实施“十三五”规划承上启下的关键一年。进入新时代，站在新起点，邢台面临难得的历史性窗口期和战略性机遇期。习近平新时代中国特色社会主义思想，为高质量发展提供了政治引领和根本遵循；省委九届六次全会确定的“抓好三件大事、打好六场硬仗、实施八项战略、深化九项改革”基本思路，为高质量发展指明了方向路径；市委九届三次全会对全市经济社会发展作出了全面部署，广大干部群众人心思进、人心思干，为高质量发展营造了凝心聚力、奋发进取的良好氛围。我们一定要以强烈的责任感、使命感、紧迫感，解放思想、转变观念，抢抓机遇、乘势而上，奋力创造无愧于时代、无愧于人民、无愧于历史的新业绩，不辜负党和全市人民的殷切期望。

　　今年政府工作总的要求是：全面贯彻落实党的十九大精神，坚持以习近平新时代中国特色社会主义思想为指导，认真落实习近平总书记对河北工作提出的一系列重要指示，贯彻落实省委九届六次全会和省“两会”精神，按照市委九届三次全会决策部署，坚持稳中求进工作总基调，坚持新发展理念，按照高质量发展的根本要求，深化供给侧结构性改革，加快建设现代化经济体系，创新竞进、协同融合、改革开放、转型升级、提质增效、改善民生、优化环境，在高质量发展中加快建设新时代经济强市、美丽邢台。

　　主要预期目标是：生产总值增长7%左右，全部财政收入增长10%以上，一般公共预算收入增长9%左右，规模以上工业增加值增长6%以上，规模以上高新技术产业增加值增长11%，固定资产投资增长7%左右，社会消费品零售总额增长10%，实际利用外资增长5%，出口总值增长5%，城镇、农村居民人均可支配收入分别增长7.5%、8%，城镇登记失业率控制在4.5%以内，万元生产总值能耗下降4.1%、用水量下降3.8%，PM2.5平均浓度下降率完成省定任务。

　　各位代表，实现上述目标，我们必须坚持以习近平新时代中国特色社会主义思想为指导，增强“四个意识”，坚定“四个自信”，切实把党的十九大精神学习成果转化为生动实践。必须坚持稳中求进工作总基调，夯实“稳”的基础，明确“进”的方向，追求“好”的目标，落实“准”的要求，把握“度”的要领，推动经济社会发展提质增效、行稳致远。必须坚持新发展理念，全面推动高质量发展，努力使创新成为第一动力、协调成为内生特点、绿色成为普遍形态、开放成为必由之路、共享成为根本目的。必须坚持开放和开发并举，城市经济和县域经济一起抓，大力发展战略性新兴产业，推动产业层次迈向中高端，努力实现开放型经济和内联式发展互促并进。必须坚持把人民利益摆在至高无上的地位，与人民同呼吸、共命运、心连心，不断满足人民日益增长的美好生活需要，让改革发展成果更多更公平惠及全市人民。

　　具体工作中，重点抓好六个方面：

　　（一）深化供给侧结构性改革，加快建设现代化经济体系。坚持质量第一、效益优先，在“破”“立”“降”上下功夫，促进增量优质、存量优化，努力实现在破中立、在消中长。

　　推动传统产业转型升级。坚定不移去产能，综合运用市场化、法治化手段，通过改造升级、减量置换、产能转移等方式，倒逼去产能行业向高端、优质、新兴产业发展。全年压减钢铁产能50万吨、煤炭66万吨，玻璃、焦炭、火电完成省定去产能任务。中钢邢机新厂区力争3月底开工建设。筹建玻璃现货交易中心。加快旭阳、建滔干熄焦改造，推进焦化、钢铁行业转型升级。

　　推动优势产业扩能提质。立足“先进装备制造和食品加工重要基地”发展定位，支持投资10亿元的君乐宝乳业基地、投资11.2亿元的今麦郎创新制造、投资18亿元的南和宠物食品产业园等项目建设，力争投资46.2亿元的玉锋大健康产业园、投资30亿元的光明生猪肉联加工项目年内开工，加强食品工业质量安全、标准化生产，从“华北粮仓”迈向“国人厨房”“世界餐桌”。全面振兴“两汽一拖”，御捷长城新能源汽车下线，红星汽车、长征汽车、北汽隆威汽车实现批量生产，邢台一拖产能、产量及市场占有率进一步提升。以工业设计推动产业流程升级、设计创新、产品优化，启动建设市级工业设计中心，培育和引入专业机构，打造“设计+”产业链。落实《中国制造2025》和“互联网+”行动，开展消费品、制造业、服务业等质量提升专项行动，积极创建“全国质量强市示范城市”。鼓励企业参与制定修订国际、国家、行业标准，培育一批标准领跑企业。支持中航上大等军民融合型企业发展，努力形成军民融合深度发展格局。

　　推动新兴产业聚集壮大。实施战略性新兴产业三年行动计划，大力发展信息技术、生物医药、新能源、新材料等高端高新产业。深化与北航VR国重、深圳易尚等校企合作，筹建信息产业大厦，打造虚拟现实、北斗导航、无人机、3D技术应用基地。投资50亿元的石药生物产业园部分投产，支持京鼎生物金银花分子医药研究院、玉锋维生素B12基地、健民土霉素原料药基地建设。投资50亿元的晶龙光伏、投资20亿元的金风科技产业园等项目尽早开工。加快中科鑫宝高分子材料、名联新材料等项目建设。大力发展现代服务业，实施消费促进、电子商务进农村进社区、商贸物流配送体系建设、实体商业转型升级四大专项行动，扶持出口百强企业和潜力较大企业发展，争创物流标准化试点城市；实施上市企业培育计划，推动晶澳太阳能回归上市，支持海裕锂能、根力多生物科技等企业上市；完善“政银保”合作机制，支持邢台银行加快发展，新引进域外金融机构1家以上。

　　推动品牌资源转化为产业优势。依托职业教育雄厚基础，深化与德国BBW教育集团、蕾娜范养老集团合作，探索推进“双元制”改革，强化产学研协同育人；支持重点院校搞好重点学科建设，鼓励大企业举办联办高质量职业教育，谋划建设食品学院、汽车学院；加强职业院校与贫困家庭对接，健全精准培训、精准扶贫、精准就业等教育扶贫机制。发挥百年眼科医院优势，支持与河北医科大学合作，扎实推进眼健康基地建设。打造国际公路自行车品牌赛事，同步办好自行车产业博览会，提升产业层级。发展智能体育，谋划建设体育小镇。办好旅发大会，全力争创5A级景区，推动旅游全域化发展，旅游业总收入增长20%以上。

　　推动县域经济做大做强。制定县域经济发展规划和激励政策，培育壮大县域特色产业，集中要素资源，一业一策制定产业转型升级路线图，推动轴承、羊绒、自行车、电线电缆、纺织服装、汽摩配件等产业增品种、提品质、创品牌，营业收入超百亿元集群达到10个以上。推进城乡建设统一规划、产业合理布局、基础设施互联互通、公共服务延伸共享。统筹城镇建设与园区发展，促进产城教深度融合。加强财源建设，提升综合实力，争取更多的县进入全省30强行列。

　　（二）加快新型城镇化进程，建设有邢台特点的魅力新城。坚持高起点规划、生态化建设、精细化管理、市场化运作，实施总投资673.6亿元的7大类191项城建重点工程，提高城市综合承载能力和服务功能，常住人口城镇化率提高2.5个百分点。

　　拉开城市主体框架。以“市域交通路网+城市主次干道+街区微循环”为主要内容，9月底前完成大外环主体工程，加快与市区道路互联互通；启动开元路南延、祥和大街等10条城市主干道，龙泉大街、北关街等19条次干道，4座桥梁新改扩建工程；整治提升20条小街巷，畅通城市路网。同时，加快邢和铁路、零换乘客运总站等建设进度，完成邢台机场主体工程，力争太行山高速建成通车，形成立体化大交通格局。

　　构建城市生态体系。以“水系+湿地+公园”为主要内容，抓好总投资22.9亿元的市区“四河”及白马河、七里河整治，开挖东部人工湖渠河道，打造环城水系；在太行山前生态脆弱区谋划建设湿地群，提升防洪能力，涵养生态水源，打造城市后花园；按照“生态修复、园中之园”的思路，依托14平方公里的中央生态公园，高水平筹办2019年河北省第三届园博会，同步谋划城市规划设计大赛，打造园博典范，形成城市绿肺，铺就“绿满邢襄、水润古城”的生态底色。

　　打造城市发展支点。以“节点街区+重点建筑+公共设施”为主要内容，启动邢东新区总投资177亿元的金融服务中心、会展中心、市民中心、工会大厦等公建项目及核心区路网建设，推动新区尽快出形象、成规模，打造京津冀协同创新与服务业承接平台；深化与大企业、大集团的战略合作，实现基础设施、产业布局同步推进。完善老城区公共服务等配套设施，改造提升体育中心、老火车站广场，完成市博物馆主馆、顺德新街、万达广场等主体工程。

　　创新城建工作机制。以“政府主导+市场运作”“EPC+PPP”为主要方式，完善城建投资模式，加快组建城建投集团，发挥各类投融资平台作用，用政府投资撬动更多社会资本。以“开春即开工”为时间节点，树立项目规划、设计和审批的效率样板，全力建设百年工程、精品工程。加强智慧城市建设，推进常态化创城，为争创全国文明城市打牢基础。

　　加快县城镇建设。坚持扩大规模和提高质量相统一、人口聚集和产业壮大相协调，扎实推进县城建设三年攻坚，着力完善城镇基础设施，完成城建投资200亿元以上，各县市至少建成1座精品公园、1个特色风貌街区。扎实推进特色小镇建设，加强古村落、古建筑、古树名木保护。全面完成数字规划系统建设，建成城镇空间数据库和信息共享平台。全部县城达到洁净城市建设标准，支持各县市区争创卫生县城、文明县城（城区）。

　　（三）大力实施乡村振兴战略，开创“三农”工作新局面。按照产业兴旺、生态宜居、乡风文明、治理有效、生活富裕的总要求，推动农业农村优先发展，做好“田园、花园、家园”文章，确保乡村振兴战略开好局、起好步。

　　坚决打好精准脱贫攻坚战。坚持聚焦再聚焦、精准再精准，全面提高脱贫质量，一般公共预算收入用于扶贫领域支出达到2%以上，确保年内4个县、124个村、4.5万贫困人口稳定脱贫。开展产业就业扶贫攻坚行动，推广“金鸡式”资产收益、“富岗式”资金入股等扶贫模式，年内实现贫困人口产业项目全覆盖。加快贫困村基础设施改造提升，实施城乡道路互通工程，改善贫困村生产生活条件和村容村貌；按照“两区同建”标准，完成临城441户、1213人易地扶贫搬迁工程，启动内丘95户、256人易地扶贫搬迁工程，确保贫困群众搬得出、稳得住、能致富。开展健康扶贫，完善贫困家庭学生救助机制，农村低保标准提高到3600元/年，提升政策兜底保障水平。对已脱贫人口，脱贫不脱钩；对新产生的贫困人口和返贫人口，及时纳入帮扶。选优配强驻村工作队，动员全社会力量，探索建立“百企帮百村”“十行包十县”“十院包千人”“十校连万户”帮扶机制。把扶贫与扶志、扶智结合起来，把救急解困和内生脱贫结合起来，引导贫困群众树立主体意识，激发摆脱贫困的内生动力。扎实开展扶贫领域腐败和作风问题专项治理，加强扶贫资金审计，确保资金使用效益。坚决落实中央和省、市委要求，坚持最硬的任务派出最精干的力量，最高的要求落实最精准的措施，最难的工作提供最强的保障，最贴近群众的使命执行最严的纪律，确保全面小康路上一个都不掉队。

　　深化农业供给侧结构性改革。实施质量兴农战略，加快推进农业由增产导向向提质导向转变，大力发展科技农业、绿色农业、品牌农业、质量农业，着力提升绿色优质品牌农产品供给能力。用产业设计的理念调整种养结构，在确保粮食安全的基础上，稳粮优经扩饲，全市粮食产能稳定在470万吨左右，推动农业大市向农业强市转变。开展农业质量年活动，健全农产品追溯体系，农产品质量检测整体合格率保持在98%以上。力争省级现代农业园区县县全覆盖，争创国家级园区。加大粮食加工转化力度，大力发展农产品加工，每个县确定1—2个优势特色主导产业，打造区域公共品牌，争创更多涉农驰名商标，年内绿色、有机和地理标志农产品认证达到100个以上。推进“互联网+现代农业”，加强各类信息技术在农业领域的应用，以大数据引导农业产业体系、生产体系、经营体系创新。实施休闲农业和乡村旅游业提升工程，推动农村一二三产融合发展，拓宽农民增收空间。

　　加快推动农村全面进步。开展农村人居环境整治三年行动，推进美丽乡村建设，深入实施“厕所革命”，加快改善水电气路讯房热等基础设施，新改建农村公路450公里，村内道路硬化率达到90%以上，实现城乡垃圾一体化处理。完善农村承包地“三权”分置制度，适度放活宅基地和农民房屋使用权，基本完成农村集体资产清产核资。创新乡村治理体系，加强农村基层基础工作，打造一支懂农业、爱农村、爱农民的“三农”工作队伍。提升农民文化素质和专业技能，培育更多新型职业农民。大力开展乡风文明建设，传承发展农村优秀传统文化，推动农村移风易俗，培育文明乡风、良好家风、淳朴民风。

　　（四）全力推进改革开放创新，做强高质量发展支撑。以更大决心和力度深化改革、扩大开放、厚植创新，为高质量发展注入强大活力和动力。

　　全方位深化各项改革。把改革作为破解发展难题的关键一招，思想再解放、改革再深入、工作再抓实，在新起点上争取新突破。持续深化“放管服”改革，扎实开展“双创双服”活动，加大向宁晋、沙河等经济扩权县市的放权力度。加快“互联网+政务服务”，实现70%以上事项“最多跑一次”，力争“一次也不跑”。坚持放管结合、并重，提升“双随机一公开”监管规范化、标准化水平。落实减税降费政策，降低企业制度性交易成本。加快国有企业战略性重组，继续深化“三供一业”改革，完善现代企业制度，强化国有资产监管，解决好改制改革企业和退市进园企业遗留问题，推动国有资本做强做优做大。深化金融财税体制改革，稳妥推进水资源税、环保税改革。优化金融生态环境，增强金融服务实体经济能力，年内完成6家农商行挂牌开业。扎实推进教育、文化、体育和医药卫生体制等改革，进一步释放改革红利。

　　加快形成全面开放新格局。以宽广的视野、包容的心态、共赢的理念，不断拓展高质量发展空间。制定完善产业链招商指导目录，发挥市级专业招商部作用，抢抓京津冀协同发展、雄安新区规划建设、2022年冬奥会等重大机遇，下大力引进科技含量高、投资体量大的产业链关联项目。增加对优质项目和项目落地开工的考核权重，提高招商实效。更深层次参与国际产能合作，借力“一带一路”支持钢铁、焦化、玻璃等优势产能“走出去”。坚持领导分包、观摩拉练、擂台赛、通报调度等机制，强化要素保障，实施市重点项目300个，年度完成投资400亿元以上，竣工投产50个以上，储备超亿元项目1000个以上。创新开发区管理运营模式，做强主导产业，突出技术研发、工业设计、检验检测等产业发展支点建设，努力形成以开发区为龙头、以重点产业聚集区为基础、以骨干企业为支撑的产业格局，力争省级开发区主营业务收入增长10%以上。

　　实施创新驱动发展战略。抓好创新主体培育，实施高新技术企业和科技型中小企业双倍增计划，年内分别新增80家、1000家以上。加大研发投入力度，设立企业创新券，探索建立科技创新企业贷款增信机制，加强知识产权创造运用保护，研发经费支出占生产总值比重达到1.25%，技术交易总额达到2.9亿元，万人发明专利拥有量达到1.12件。加强创新平台建设，新增省级科技创新平台10家、市级100家，新建省级院士工作站2家。鼓励创业带动就业，新增市级以上孵化器2家、众创空间5家以上。加强高层次人才和创新团队引进，实施“百名硕博引进计划”，为高质量发展提供智力支撑。

　　（五）打好污染防治攻坚战，加快营造良好人居环境。像对待生命一样对待生态环境，着力解决突出环境问题，让人民群众切实感受到环境改善的成果。

　　狠抓大气污染综合治理。制定蓝天保卫战三年作战计划和冬季清洁取暖三年规划，全面推进“保底线、退后十”集中攻坚。实施革命性工程，打好产业结构、能源结构、运输结构调整组合拳，持续提升人民群众的蓝天获得感。开展工业企业全面达标排放行动，抓好重污染企业搬迁改造、错峰生产、错峰运输，巩固“散乱污”治理成效。抓好清洁能源替代，发展节能环保产业，有序推进清洁供暖改造，拆除35蒸吨及以下燃煤锅炉，压减煤炭消费56万吨以上。强化面源污染精细化管控，“以克论净”加强城市道路管理，通过大外环和零换乘客运总站，实现每天约2万辆客货车远端绕行；规划新建4个企业铁路专线，全市铁路货运比提高40%以上。提高科学治霾水平，谋划设立京津冀合作治霾技术研发推广中心，探索无人机影响天气作业。扎实做好油气回收治理、老旧机动车淘汰、秸秆禁烧和综合利用等工作。

　　开展水污染集中治理攻坚。全面落实河长制，加快大沙河综合治理，推动7条重污染河流退污还清，开展纳污坑塘专项整治，城市黑臭水体消除80%以上，地表水劣Ⅴ类水体断面比例控制在75%以内。加强饮用水源地保护，开展饮用水源地保护区违规项目排查整治和集中式饮用水源地水质监测评估。完成市污水处理二厂建设和七里河污水处理厂提标改造，年底前市区基本实现雨污分流、污水处理达标两个百分之百；加强县乡污水处理厂建设，实现省级工业园区、县城和重点乡镇污水集中处理全覆盖，县城污水处理率达到88%以上，污泥处理项目提标改造全面完成。

　　加强生态保护和修复。统筹山水林田湖草系统治理，完成农用地土壤污染情况详查，抓好新河县土壤污染治理与修复试点，市、县生活垃圾无害化处理率分别达到96.6%以上、92.3%以上。弘扬塞罕坝精神，启动实施国土绿化三年行动，用好国家储备林贷款，推进国家森林城市创建，力争完成造林绿化面积50万亩以上。持续开展生态修复，启动生态红线区域勘界定标。全面做好地下水压采、南水北调江水消纳、露天矿山综合整治等工作。同时，坚持督企督政并重，实行最严厉的监管执法，坚决打击环境违法违规行为。

　　（六）坚持以人民为中心，增强群众获得感幸福感安全感。始终把人民群众的小事当作自己的大事，一件事情接着一件事情办，一年接着一年干，用点点滴滴的变化，汇聚成邢台的新形象。坚持尽力而为、量力而行，今年重点实施20件民生实事，逐个制定实施方案、保障措施，确保高标准、高质量按时完成。

　　大力发展社会事业。以办人民满意的教育为目标，继续推进市区基础教育三年攻坚，实施特殊教育提升计划，创新高中办学体制，促进城乡义务教育一体化发展，全市新增标准化学校100所以上。实施文化强市战略，建立健全市县乡村公共文化服务设施网络，加强文化产业示范园区和示范基地建设，推进大运河文化带规划建设，实施邢襄文化弘扬工程，加强文物保护利用和文化遗产保护传承，做好地方志史编修利用工作，创作更多具有邢台特色的新时代文艺精品。开展全民健身活动，完善基层公共体育设施，推进县级全民健身活动中心、乡村农民体育健身、城市社区15分钟健身圈等工程建设，全面提高人民身体素质。

　　构建多层次社会保障体系。加强全方位就业服务，深入实施高校毕业生就业创业促进计划和基层成长计划，扎实做好去产能分流职工、城镇就业困难人员等重点群体安置工作，年内新增城镇就业6.2万人。支持农村劳动力转移就业和农民工返乡创业，坚决治理拖欠农民工工资行为。推进养老保险提标扩面，深入实施全民参保计划，扩大民营企业、农民工、灵活就业人员覆盖范围。加强健康邢台建设，推进“三医联动”，落实药品采购“两票制”，抓好医联体、医共体、专科联盟建设，推行分级诊疗制度，扩大家庭医生签约服务范围，推广“医养结合”模式和巨鹿长期护理险办法，继续做好计划生育工作。着力保障群众基本住房需求，建立健全多主体供给、多渠道保障、租购并举的住房制度，发展住房长期租赁市场，加大公租房保障力度。

　　打好防范化解重大风险攻坚战。有效防控金融风险，理顺金融监管体制，实施全过程、全覆盖、穿透性监控，严厉打击非法集资、违规融资担保、恶意逃废债等行为，建立防范化解金融风险长效机制，通过“监控+预警+处置”有效联动，坚决守住不发生系统性、区域性金融风险底线。有效防控政府性债务风险，增强预算编制科学性、标准化，落实地方政府债务规模限额管理，抓好债务存量化解和增量控制，加强对政府投融资平台、产业投资基金、PPP、政府购买服务等领域的规范化管理，坚决制止变相违规举债。有效防控安全生产风险，持续深化煤矿及非煤矿山、危险化学品、道路交通、油气管网等重点行业领域专项整治，坚决遏制重特大安全生产事故；开展“千场消防安全大宣讲”，完善消防基础设施；加强食品药品全过程监管，争创国家食品安全示范城市。有效防控社会风险，完善社会治安防控体系，构建新型矛盾排查化解常态化机制；依法规范信访秩序，及时有效解决群众合理诉求；强力开展扫黑除恶专项斗争，重拳打击黑恶痞霸势力；严厉打击涉枪涉爆、电信诈骗等违法犯罪行为，及时妥善处置各类突发事件，保障人民群众生命财产安全。

　　加强全民国防教育、国防动员和后备力量建设，大力支持驻市部队推进新时代强军事业，着力解决随军家属就业和子女入学问题，做好军队退役人员安置和管理服务工作，巩固军政军民团结的好局面。充分发挥工会、共青团、妇联等人民团体桥梁纽带作用。继续做好民族宗教、外事侨务、新闻广电、统计档案、邮政通信、人防地震、老龄妇幼、残疾人事业、援疆援藏等工作。三、努力建设人民满意政府新时代呼唤新作为。我们必须深入学习宣传贯彻党的十九大精神，忠诚履职、勤勉尽责，同全市人民想在一起、干在一起，以实干回报人民的期望。

　　（一）牢固树立“四个意识”，坚定正确的政治方向。旗帜鲜明讲政治，坚决捍卫习近平总书记的核心地位，在政治立场、政治方向、政治原则、政治道路上同以习近平同志为核心的党中央保持高度一致。切实提高政治站位，把对党忠诚、为党分忧、为党尽职、为民造福作为根本政治担当，推进“两学一做”学习教育常态化制度化，扎实开展“不忘初心、牢记使命”主题教育。坚持把党对一切工作的领导贯彻到政府工作各个领域、各个方面，确保中央大政方针和省、市委决策部署落地生根。

　　（二）坚持严格依法行政，让权力在阳光下规范运行。弘扬宪法精神、增强宪法意识，自觉尊法崇法学法用法，始终把法治思维贯穿政府决策、执行、监督全过程，严格执行重大行政决策、规范性文件制定法定程序，实现科学民主决策。严格规范公正文明执法，推动综合执法改革，加大关系群众切身利益的重点领域执法监督力度。推进政务公开，自觉接受人大及其常委会的法律监督、工作监督和政协的民主监督，重视公众监督和舆论监督，加强法纪监督和审计监督，确保各项工作在法律框架内开展。

　　（三）强化担当狠抓落实，全面提高政府工作效能。不驰于空想、不骛于虚声。加强学习和实践，培养专业能力，弘扬专业精神，找准短板弱项，落实落实再落实，提高新时代把握和推动高质量发展的能力和水平。进一步精简会议和文件，开务实的会、发管用的文、办有效的事。对群众关注的事，定一条是一条，条条算数；对群众期盼的事，干一件成一件，件件落实。坚持问题导向和目标责任导向，实干实政，善作善成，年初建账、年中查账、年底算账，以工作“晾晒”倒逼工作落实，以“钉钉子”精神做实做细做好各项工作。

　　（四）大力优化营商环境，打造邢台温度邢台态度邢台速度。企业家是财富的创造者、创新活动的实践者。尊重和支持企业家，加强企业家队伍建设，激发和保护企业家精神，构建亲清新型政商关系，是广大公务员的职责所在。要支持本地企业家热爱家乡、发展家乡，欢迎外地企业家投资邢台、共谋发展，集中整治“庸政、懒政、怠政”行为和“不作为、慢作为、乱作为”“既不点头、也不摇头”现象，使广大企业家感受到亲商、安商、富商的邢台温度和邢台态度。探索建立营商环境评价体系，保护企业合法权益，让各类市场主体创新创造的活力竞相迸发，成就高质量发展的邢台速度。

　　（五）持之以恒正风肃纪，推动干部清正政府清廉政治清明。严守政治纪律和政治规矩，带头贯彻落实中央八项规定及其实施细则精神，严格执行省、市有关规定，坚决纠正“四风”突出问题，重点整治形式主义、官僚主义新表现。认真落实全面从严治党要求，履行主体责任和“一岗双责”，加强政府系统廉政建设和反腐败工作，对群众身边“微腐败”现象对症下药，从严查处，绝不姑息。政府系统广大公务员要知敬畏、存戒惧、守底线，持廉守正，干干净净地为邢台人民办实事。

　　各位代表，新的历史方位，赋予我们新的历史担当。让我们更加紧密地团结在以习近平同志为核心的党中央周围，以习近平新时代中国特色社会主义思想为指导，在市委的坚强领导下，团结依靠全市人民，以永不懈怠的精神状态和一往无前的奋斗姿态，走新路、有作为、创亮点、守底线，奋力开创新时代全面建设经济强市、美丽邢台新局面！
